# The Role of Radiation Therapy in the Treatment of Non-Melanoma Skin Cancer

**DOI:** 10.3390/cancers15092408

**Published:** 2023-04-22

**Authors:** Eyal Yosefof, Noga Kurman, Dan Yaniv

**Affiliations:** 1 Department of Otorhinolaryngology Head and Neck Surgery, Rabin Medical Center, Petah Tikva 4941492, Israel; 2 Sackler Faculty of Medicine, Tel Aviv University, Tel Aviv 4941492, Israel; 3 Institute of Oncology, Davidoff Center, Rabin Medical Center, Petah Tikva 4941492, Israel; 4 Department of Head and Neck Surgery, MD Anderson Cancer Center, Houston, TX 77054, USA

**Keywords:** non-melanoma skin cancer, cutaneous squamous cell carcinoma, cutaneous basal cell carcinoma, radiotherapy, chemotherapy, immunotherapy

## Abstract

**Simple Summary:**

While surgery remains the main treatment modality for non-melanoma skin cancer (NMSC), radiotherapy still plays a major role in the treatment algorithm, even with the emergence of immunotherapy and the revolution it is causing in treatment paradigms. The aim of our review is to describe the role of radiotherapy in the definitive and adjuvant setting for NMSC. We also aim to describe the role of radiotherapy combined with immunotherapy in these cases.

**Abstract:**

Non-melanoma skin cancer (NMSC) is the most common malignancy in the United States. While surgery is considered as the main treatment modality for both cutaneous basal cell carcinoma (cBCC) and cutaneous squamous cell carcinoma (cSCC), radiotherapy plays an important role in the treatment of NMSC, both in the adjuvant setting for cases considered high-risk for recurrence, and in the definitive setting, when surgery is not feasible or desired by the patient. The last years have seen the emergence of immunotherapy treatment for cases of advanced cSCC in the palliative, and possibly neoadjuvant settings, making the treatment paradigm more complex. In this review, we attempt to describe the different radiation modalities available for the treatment of NMSC, the indications for adjuvant post-operative treatment with radiotherapy for cSCC, the role of radiotherapy in elective neck treatment, and the efficacy, safety, and toxicity profile of this treatment in these different settings. Furthermore, we aim to describe the efficacy of radiotherapy combined with immunotherapy as a promising horizon for treating advanced cSCC. We also aim to describe the ongoing clinical studies that attempt to examine future directions for the role of radiation treatment in NMSC.

## 1. Introduction

Non-melanoma skin cancer (NMSC) is the most common malignancy in the United States, accounting for over three million new cases each year. A total of 80% of them are basal cell carcinoma (BCC), 20% squamous cell carcinoma (SCC), and less than one percent other tumors such as Merkel cell carcinoma and adnexal tumors [1,2]. The risk factors for NMSC mainly include high cumulative ultra-violet radiation sun exposure [3,4], especially to the head and neck area; increasing age; immunosuppression—either drug-induced among organ transplant patients and cased by an autoimmune disease or due to hematologic malignancies, specifically chronic lymphocytic leukemia (CLL) or human immunodeficiency virus (HIV); chronic inflammation [5]; and certain genetic conditions such as Xeroderma Pigmentosum [6].

While the standard of care for curative intent therapy is surgical treatment for both cutaneous BCC (cBCC) and cutaneous SCC (cSCC) patients, radiotherapy plays a significant role both in the definitive and adjuvant settings [7].

In the definitive setting, radiotherapy is considered an accepted alternative approach to surgery among patients who are medically inoperable, those who refuse surgery, and in cases where surgical excision may be associated with a poor cosmetic outcome [8]. 

Different radiation methods—including Kilo-voltage (soft) X-rays, mega-voltage electrons, mega-voltage X-rays, and low dose rate (LDR)/high dose rate (HDR) interventional radiotherapy (brachytherapy) and proton therapy—are all accepted radiotherapy modalities for NMSC [9].

In the adjuvant setting, radiotherapy is considered by the National Comprehensive Cancer Network (NCCN) in cases of positive tumor margins after surgical resection among patients not amenable to re-excision, and in cases with high-risk features for tumor recurrence, such as peri-neural invasion (PNI), lympho-vascular invasion (LVI), head and neck location, ill-defined borders, a rapidly growing tumor, a tumor larger than two centimeters, deep tumor invasion beyond the dermis or into deep structures (i.e., bone invasion), specific histologic features such as acantholytic, adenosquamous, metaplastic, or desmoplastic subtypes, recurrent tumors, and tumors in immunosuppressed patients [10]. 

The aim of our review is to explore and describe the different radiation techniques used for NMSC, the relevant literature regarding the role and indication for radiotherapy treatment for cBCC and cSCC, both in the definitive and adjuvant setting, and to study its current role in elective neck treatment among these patients. Furthermore, in the evolving world of immunotherapy, we aim to investigate whether the emergence of Hedgehog pathway inhibitors for the treatment of advanced-stage cBCC and of immune checkpoint inhibitors (such as anti PD-1 antibodies, namely cemiplimab and pembrolizumab) as a treatment modality for advanced cSCC may alter the role of radiation therapy in these cases.

## 2. Methods

We performed a literature review and searched the Pubmed/Medline database for studies within the scope of our review, published until 31 December 2022, including the strings: “radiotherapy for cutaneous squamous cell carcinoma”, “radiotherapy for cutaneous basal cell carcinoma”, “radiotherapy for non-melanoma skin cancer”, “radiation treatment for cutaneous squamous cell carcinoma”, “radiation treatment for cutaneous basal cell carcinoma”, and “radiation treatment for non-melanoma skin cancer”. Each study was independently reviewed by two authors (E.Y. and D.Y) for relevance to the study. In cases of disagreement, a third author (N.K.) made the final decision regarding the article’s relevance to the review. 

## 3. Radiotherapy Techniques for NMSC

### 3.1. Early-Stage Lesions

**Figure 1:**
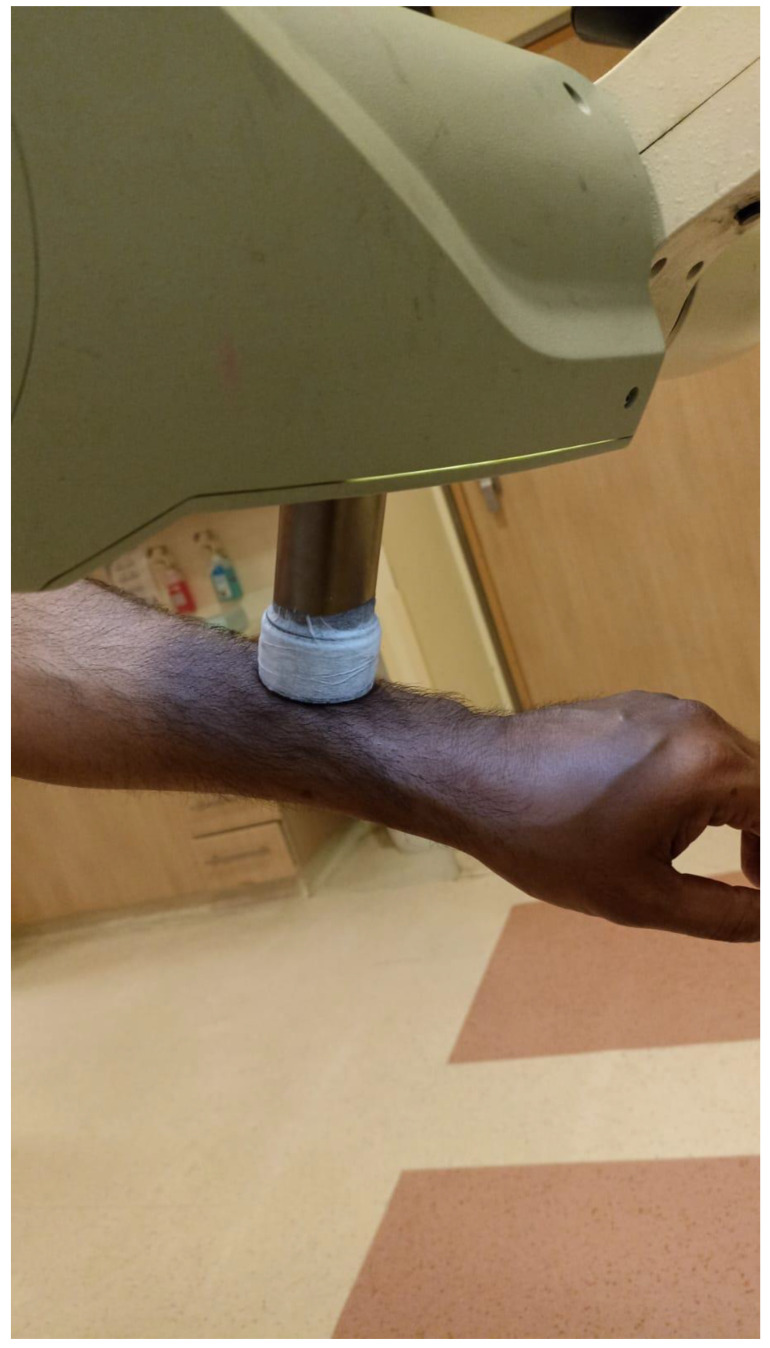
Soft X-ray cone positioning.

**Figure 2:**
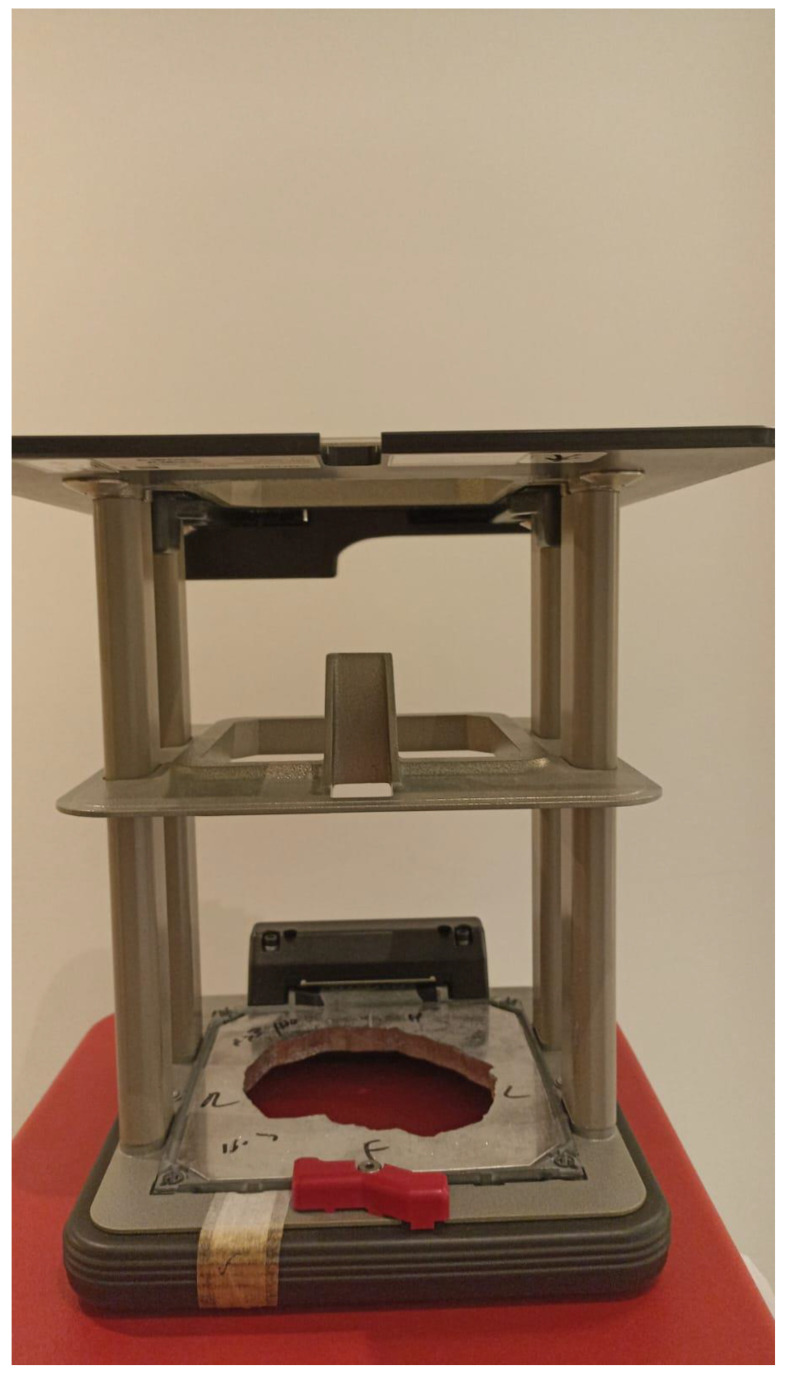
Personalized lead block for electron treatment.

**Figure 3:**
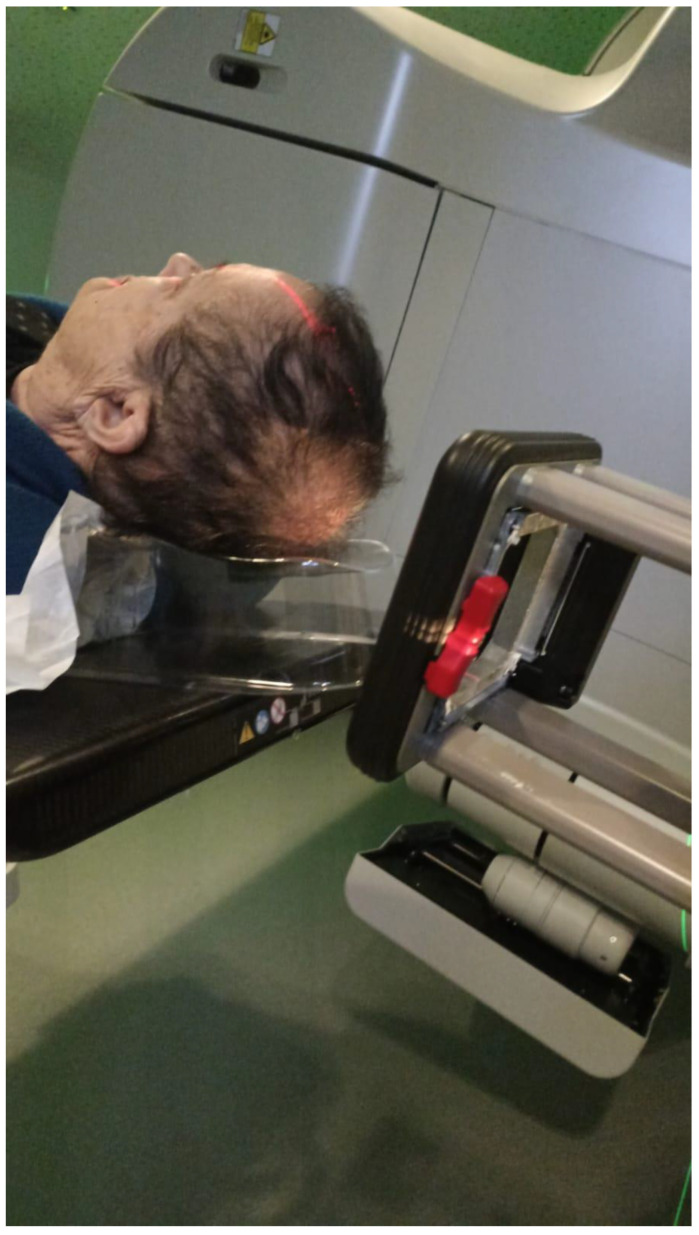
Electron beam treatment.

**Figure 4:**
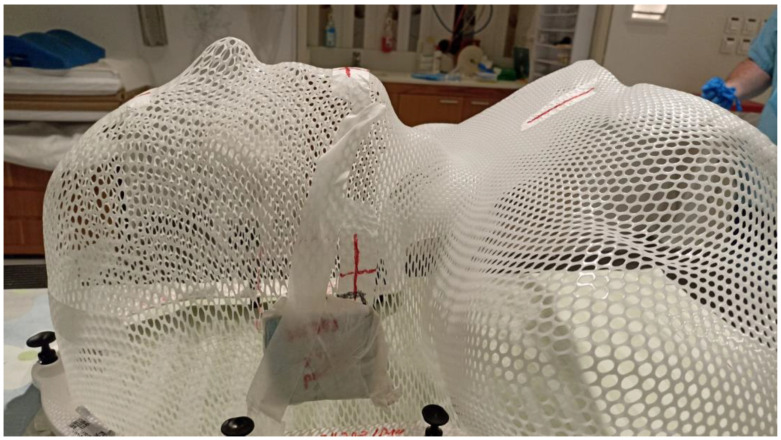
Thermoplastic mask with personalized bolus attached for MV photon treatment.

**Figure 5:**
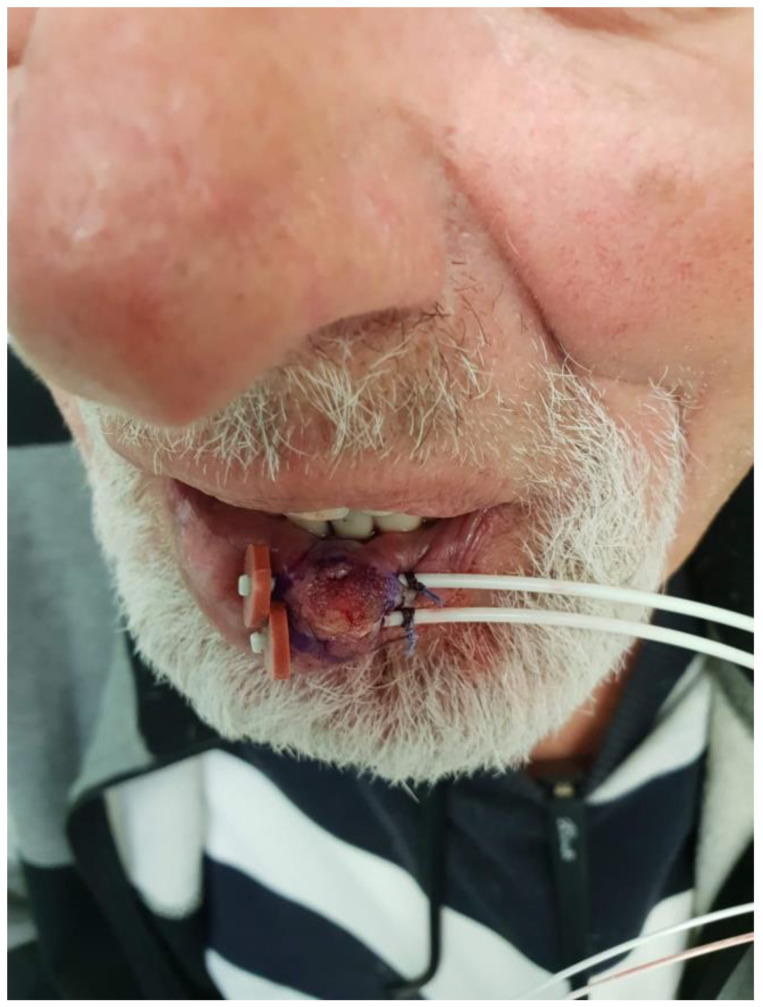
Interstitial catheters for lip interventional radiotherapy (brachytherapy).

In cutaneous lesions, a major challenge is achieving a therapeutic dose at the surface while targeting the tumor to its entire depth. Several methods may be employed:

1. Soft X-ray (contact) therapy: This method entails placing a cone directly onto the irradiated surface, typically with the delivery of the dose at energies of 30–100 kV. This may target lesions up to 10 mm deep at a therapeutic dose. The advantages of this method include a low penumbral dose and easy clinical setup. Its major disadvantages, especially in the definitive setting, are unclarity regarding the subclinical spread, and technical difficulty in measuring the tumor depth. This method may be preferred in well-demarcated and well-palpable superficial and symmetrical lesions [11] (Figure 1).
2. Electron beam radiotherapy: This is delivered via a linear accelerator (LINAC) at energies of 6–20 MeV and may target superficial lesions, with a therapeutic depth of up to 5 cm (which is the depth of the 90% isodose line for 20 MeV). The setup may be clinical or assisted by computerized tomography (CT) simulation; beam collimation is performed by lead blocks (standard or personalized). This method is useful in treating relatively large fields and lesions deeper than 1 cm without compromising superficial tissues. Its disadvantages include a relatively large lateral spread (especially at higher energies), cumbersome beam collimation, and a skin sparing effect at 6 MeV [12] (Figure 2 and Figure 3).
3. Mega-Voltage (MV) photon beam therapy: This is delivered via a LINAC at energies of 6–18 MV. A clinical setup is possible, but treatment is mostly planned via CT simulation. This method allows for more sophisticated treatment planning (forward or inverse) and beam collimation, and higher certainty regarding the dose delivery. However, MV photons have a skin sparing effect, and therefore require a tissue compensator (bolus) to be placed on the superficial portion of the tumor, which may be technically challenging and reduce the aforementioned setup certainty. This is the preferred method for the treatment of tumors with a deep-set component or with proximity to critical structures [13] (Figure 4).
4. Interventional radiotherapy (brachytherapy): This is currently mostly performed with interstitial catheters and an HDR source, or by personalized surface molds. This method is useful for lesions with complex geometry, where external beam therapy may result in an inhomogeneous dose distribution, or in proximity to critical structures; this method may also be employed after failure of conventional EBRT due to the ability to deliver high doses per fraction with little collateral damage. While data regarding the specific protocols are limited, common protocols for HDR include 30–50 Gy given in 5–10 fractions [14] (Figure 5).
5. Proton therapy: The use of this novel technique of charged particle therapy is becoming more and more widespread in routine practice, especially in the setting of re-irradiation. It is delivered via a particle accelerator and generally planned inversely. Its major advantage is the Bragg peak, which delivers a high dose to the target with a very rapid falloff, and therefore a very low exit dose. Its main disadvantages are the cost and scarce availability, as well as technical challenges regarding the entrance dose [15,16].

### 3.2. Advanced Lesions—Locally Advanced or Neck Involvement

Radiotherapy to deep-set structures, whether in the definitive or the postoperative setting (as either adjuvant or salvage treatment), is usually performed by MV photon intensity modulated radiotherapy (IMRT) with strict immobilization of the patient (in the head and neck region, via a thermoplastic mask). This allows for the protection of normal structures while delivering a homogenous dose to the target.

An alternative is intensity modulated proton therapy, which may protect normal structures better and reduce the integral dose due to a very low exit dose [15].

### 3.3. Dose Fractionation Schemes

A wide range of doses and fractionation schemes are utilized clinically, as is reflected in the NCCN guidelines, recommending that a biologic effective dose of 70–93 Gy is to be achieved in the definitive setting and 60–79 Gy in the adjuvant setting, referring to an alpha/beta ratio of 10 [10].

Hypofractionation may be useful for small-volume superficial lesions, where it may not result in severe toxicity [17,18]. The NCCN guidelines recommend a biologic effective dose of 56–88 Gy in the definitive setting, and 56–70 Gy in the adjuvant setting. Hypofractionation of large volumes (i.e., neck radiotherapy) is not formally recommended [10].

In early-stage lesions, the treated volume is typically the gross tumor with 0.3–0.5 cm of clinical target volume.

Elective neck irradiation: When treating locally advanced lesions with definitive or adjuvant intent, elective neck radiotherapy may be employed. Commonly, the radiation fields are large and may include (depending on the location of the primary) levels 2–4, the superficial lobe of the parotid gland, level 1B for lesions anterior to the ear, and level 5 for posterior lesions. Contralateral elective radiotherapy is generally not warranted [19].

## 4. Radiation as Primary Therapy with Curative Intent

While surgery remains the standard-of-care curative intent treatment modality for cBCC and cSCC, primary radiotherapy is considered as an acceptable alternative among patients who are not fit for general anesthesia (due to older age or co-morbidities), in cases where surgical resection may be associated with poor cosmetic and functional results such as the midface area, and when patients refuse surgical treatment [2,20]. 

Regarding BCC, a study by Kwan et al. [21] retrospectively reviewed the results of 61 patients with both early and advanced stage (T2 or higher) cutaneous BCC treated by definitive radiotherapy and demonstrated an 86% four-year locoregional control rate, with 100% disease-specific survival. These high control rates were also demonstrated in a different study by Locke et al. [22] with a 92% local control rate among 389 patients treated with radiotherapy for BCC, and 92% of patients reporting excellent cosmetic results as well. While these studies show the high local control rate achieved by definitive radiation, they do not compare radiotherapy with surgery and did not stratify the tumors by early/advanced stage. 

### 4.1. Early-Stage cBCC and cSCC

A randomized trial by Avril et al. [23] compared surgical treatment with radiation treatment for patients with cutaneous BCC of the face measuring up to 4 cm. They were able to demonstrate a significantly higher rate of local control among the 174 patients treated with surgery compared with the 173 treated with radiation (Log rank *p* = 0.003). While 87% of patients treated with surgery reported a good cosmetic result, only 69% of the radiotherapy arm reported such result (*p* < 0.01).

Regarding definitive radiotherapy for early cSCC, a study by Cognetta et al. [24] followed 994 cSCC patients who were treated by definitive intent superficial X-ray radiation for early stage T1-2 tumors, with a 5-year local control rate of 94.2%. A similar study by Barysch et al. [25] described an 86% 5-year control rate among 180 lesions treated with superficial radiotherapy. They demonstrated an association between the tumor histological grade and recurrence, as poorly differentiated tumors were significantly more likely to recur, compared with well/moderately differentiated tumors. These studies and several other studies discussing early-stage cBCC and cSCC treated with radiotherapy are described in Table 1.

### 4.2. Advanced Stage Non-Metastatic cSCC and cBCC

Lee et al. [28] studied the role of radiotherapy as a definitive treatment modality among patients with advanced cSCC. They demonstrated a 53% initial 5-year control rate and 74% ultimate control rate (including patients with treatment failure successfully salvaged with surgery). Bone involvement, perineural invasion, and prior radiotherapy were all associated with a worse control rate. Al-Othman et al. [29] also evaluated the efficacy of definitive radiotherapy for advanced-stage NMSC, and found a 53% initial and 90% ultimate control rate, quite similar to the results of Lee. As the performance of CT simulation for treatment planning was not available during the time period described, the relatively low primary radiation treatment control rate described in the abovementioned papers is not surprising, as marginal failure was more common in those since CT simulation for treatment planning was not yet available. 

PNI was introduced into the 8th edition of the AJCC staging for SCC and non-Merkel NMSC as a defining criterion for a T3 tumor [30]. PNI was not a part of the 7th edition [31], and this modification emphasizes its prognostic importance. Balamucki et al. [32] described the role of definitive radiotherapy in cases of cSCC with clinical PNI—defined as cases of PNI identified by either the patient’s symptoms or findings in their physical examination, or radiological evidence of tumor involvement along the nerve, usually either the Trigeminal or Facial nerve. A total of 36 patients were treated with RT alone, with a 5-year local control rate of 42%, and 13 with chemoradiotherapy (CRT), with a 5-year 62% local control rate. Studies regarding definitive radiotherapy for advanced-stage cBCC and cSCC are described in Table 2. 

It appears that definitive radiotherapy is a reasonable alternative to surgery for early-stage cBCC and cSCC, and even in advanced-stage tumors, with a possible need for surgical salvaging in cases of disease recurrence. 

Regarding cBCC that is not amenable for curative intent surgery or radiotherapy (mostly in the non-curative locally advanced setting, as metastatic cBCC is extremely rare), the use of the Hedgehog pathway inhibitors Vismodegib and Sonidegib was demonstrated to result in a local control rate of 60–80% in different studies [1,33]. A durability in the response between 7.6 months and over a year was described [34,35]. This treatment modality enables sparing, or at least delaying the need for extensive and possible re-irradiation of recurrent cBCC cases.

**Table 2:** Summary of articles describing the role of radiotherapy in the definitive setting in advanced cutaneous basal and squamous cell carcinoma.

Author	Number of Patients	Pathology	Outcome	Complications	Radiotherapy Modality and Fractionation
Lee 1993 [28]	67	T4 BCC + SCC	5 years local control with radiotherapy—53% With salvage surgery 5 years—74%	9% severe complication	X-ray 250 kV—31.3% External beam—28.4% Megavoltage photons—25% Electron beam—12% Other modality—3%
Kwan 2004 [21]	182	T2 or higher BCC + SCC	4 years local control for BCC—86% 4 years local control for SCC—58%		Orthovoltage X-ray, electron beam, megavoltage photons
Locke 2001 [22]	65	T3/T4 BCC + SCC	5 years local control for BCC—85% 4 years local control for SCC—71%	5.8%	Soft X-ray (contact) radiotherapy—60% Electron beam radiotherapy—19% Soft X-ray and electron beam combination—20% Megavoltage—<2%
Al-Othman 2001 [29]	88	Stage 4 BCC + SCC	5 years local control with radiotherapy—53% With salvage surgery 5 years—90%	17% severe complication	External beam—87.5% Interventional radiotherapy (brachytherapy)—2.2% External beam with interventional radiotherapy (brachytherapy) boost—10.3%
Balamucki 2012 [32]	49	SCC with incidental PNI	RT 5 years local control—42% CRT 5 years local control—62%		No data regarding radiotherapy modality
Kim 2018 [36]	34	T3/T4 BCC + SCC	3 years DSS for BCC—93.3% 3 years DSS for SCC—38.3%		Orthovoltage photons Megavoltage photons Electron beam therapy Proton therapy
Hiura 2019 [37]	21	Stage 4 SCC	One year overall survival—79% One year progression-free survival—44%		Chemoradiotherapy—no specific radiation protocol described
Hazim 2021 [38]	21	SCC with clinical PNI	RT/CRT 2 years local control—59.8%	Dermatitis—67% Mucositis—57%	External beam radiotherapy—52% Proton therapy—48%

## 5. Adjuvant Treatment

The NCCN guidelines [10] recommend consideration of adjuvant radiotherapy in cases of positive margins after surgical resection, especially when re-resection is not feasible. The consideration of adjuvant radiation is also recommended in cases with “extensive perineural, large (>0.1 mm) or named nerve involvement” and in cases of regional spread. Adjuvant radiotherapy is also considered when high-risk features are present, such as a primary location in the head and neck region, hands or feet; poorly defined borders; deep tumor invasion; recurrent tumor; poorly differentiated histology; lympho-vascular spread; and several other features. We aim to describe the relevant and contemporary literature overviewing these indications. 

### 5.1. Perineural Invasion

PNI is more frequently seen in patients with cSCC (5% to 10%) than in patients with BCC (2% to 5%) [39]. In the case of clinical PNI (cPNI), the Trigeminal (V) and Facial (VII) cranial nerves (CN) are commonly affected, typically involving retrograde progression (progression of the disease from the periphery (skin) toward the brain/brainstem).

PNI is considered a factor associated with a worse prognosis among cSCC, as it is associated with higher rates of local, regional, and distant recurrence [40,41,42].

Patients with asymptomatic microscopic PNI may benefit from local adjuvant radiotherapy over a wide field but do not necessarily require treatment to the entire course of the relevant cranial nerve [13].

Harris et al. [43] retrospectively reviewed 349 patients with head and neck cSCC who were treated by surgery for either an early- or advanced-stage tumor. Of them, 191 patients (54.7%) received adjuvant radiotherapy and 158 (45.3%) were treated by surgery without adjuvant treatment. Patients with PNI, a regional disease, poorly differentiated tumors, and an immunosuppression background were more likely to be treated by both surgery and radiation therapy. In a multivariate analysis, radiotherapy was associated with an improved overall survival (OS) (HR 0.59, 95% CI 0.38–0.9). Furthermore, in a sub-set analysis of patients with PNI, adjuvant radiation was associated with better DFS (disease-free survival) and OS (HR 0.47, 95% CI 0.23–0.93 and HR 0.44, 95% CI 0.24–0.86, respectively). A similar analysis of patients with regional spread also demonstrated an advantage for adjuvant radiation therapy among this sub-group for both DFS and OS (HR 0.36, 95% CI 0.15–0.84 and HR 0.3, 95% CI 0.15–0.61, respectively). No similar association was demonstrated regarding T3/T4 tumors or poorly differentiated malignancies. 

A study by Stevenson et al. [44] attempted to evaluate the role of adjuvant radiotherapy in cSCC with perineural invasion. A cohort of 31 patients, all with a large-caliber PNI (>0.1 mm in diameter), or small-caliber PNI with other high-risk features were referred to adjuvant radiotherapy. Out of them, only 15 (48.4%) completed the treatment, with patients’ refusal being the main reason for waiving of the treatment among the other 16 patients. The two treatment groups were homogenous other than an older mean age among the non-adjuvant treatment group (age over 70—66.7% of non-adjuvant group vs. 33.3% among adjuvant radiotherapy group, *p* = 0.04). The five-year DFS was significantly improved among patients who received adjuvant radiation compared with those who were not treated (100% vs. 68.8%, Log-rank *p* = 0.01). 

Gluck et al. [40] described the patterns of failure among patients with cSCC with PNI. The series included 11 patients with clinical or radiological PNI treated with both surgery and radiotherapy. The analysis demonstrated that recurrences could appear along the original nerve involved, but also along other nerves—for example, patients with CN VII involvement of the original tumor who failed along the V3 branch of the Trigeminal nerve, or vice versa. The authors hypothesized that a possible mechanism is the communication between CN V and CN VII at the auricoulotemporal and Greater Superficial Petrosal nerve. Hence, they recommended that clinical PNI cases should be irradiated proximally along the involved nerve course up to the skull base when feasible. Furthermore, they also recommended elective irradiation along the course of microscopically involved nerves, as cases not irradiated in their cohort eventually demonstrated clinical PNI. 

Several other publications also described the prognosis of PNI among cSCC and demonstrated a favorable prognosis when adjuvant radiotherapy was offered [45,46,47].

Bryant et al. [48] described a cohort of 26 patients with NMSC with PNI treated with proton therapy, either in the definitive (73%) or adjuvant (27%) setting. The treatment protocol included a total dose of 70 Gy given in 1.8 to 2 Gy per fraction, or hyperfractionation proton therapy given at 1.2 Gy in 2 daily doses to a total dose of 72 to 74.4 Gy. The latter protocol was usually practiced when optic structures were considered close to the planning target volumes. The involved nerves were irradiated to either the skull base, nerve root, or ganglion, according to the deepest invasion of tumor as viewed upon imaging. The 3-year loco-regional control and OS rate were 65% and 59%, respectively. Thirteen patients (50%) experienced a grade 3 or higher treatment-related toxicity, including keratitis and brain necrosis in 4 patients each (15%). 

Despite the fact that the publications described above all favored adjuvant radiotherapy for PNI, the addition of radiation treatment is not consensual and the NCCN guideline only recommend a multidisciplinary consultation and the consideration of adjuvant radiotherapy in the case of PNI involving large or named nerves [10]. 

### 5.2. Regional Disease

A study by Coombs et al. [49] reviewed 63 patients with metastatic cSCC to the parotid. All patients underwent surgical therapy that included parotidectomy with neck dissection. Fifty-one patients (81%) were treated with adjuvant radiotherapy. The five-year disease-specific survival was significantly improved among patients treated with adjuvant radiotherapy (84% vs. 48%, *p* = 0.0076). A similar study by Hirshoren et al. [50] also describes a series of 78 patients with parotid cSCC metastases. Sixty-five underwent superficial parotidectomy (83.3%) and 13 total parotidectomy (16.7%). Of note, the median age of the patients was 79 years. Sixty-four patients were treated with adjuvant radiotherapy (82.1%). The institute’s protocol for adjuvant radiation included 60 Gy in 30 fractions, or 66 Gy in 33 fractions in cases of extracapsular extension or positive margins. The authors state that patients who were medically compromised or had poor performance status were treated with a hypofractionation regimen, although they do not state how many patients received which protocol. Patients treated with superficial parotidectomy and adjuvant radiation demonstrated an improved 5-year OS compared with patients treated by surgery alone (50% vs. 20%), and a better 2-year regional control (89% vs. 40%). Among patients who underwent superficial parotidectomy and adjuvant radiation, the ipsilateral regional recurrence rate was 9% (5 out of 54), compared with 37% (4 out of 11) among patients who underwent superficial parotidectomy without adjuvant radiotherapy. 

Veness et al. [51] described 74 patients with cSCC with cervical (non-parotid) metastases and compared 52 patients treated with surgery (either selective or comprehensive neck dissection) and adjuvant radiotherapy, 13 patients treated with surgery alone, and 9 patients treated with radiotherapy alone, either due to a surgically inoperable disease or significant co-morbidities defining them as medically inoperable. The median dose delivered in the adjuvant setting was 60 Gy in 2-Gy daily fractions, while in the definitive setting the median dose was 66 Gy, also in 2-Gy fractions. Among 22 patients (36% of all the radiation-treated patients), the radiation field included all or part of the parotid, except for the neck. While no significant difference was demonstrated regarding the OS, the 5-year DSS was significantly improved among patients treated with both modalities, compared with patients treated with radiotherapy or surgery alone (75% vs. 52% vs. 18%, Log-rank *p* = 0.01). 

These studies emphasize the importance of adjuvant radiation among patients with regional spread, either to the parotid or to the neck. 

### 5.3. Immunosuppression

Immunosuppression is considered a risk factor for NMSC, with solid organ transplant recipients experiencing a 65- to 100-fold increase in the incidence of cSCC [52,53,54,55]. Furthermore, it seems that cSCC among this patient group is more aggressive in nature, with a higher rate of locoregional recurrence and distant metastases [56,57,58]. CLL patients are also prone to a more aggressive course of cSCC [59].

Kadakia et al. [60] evaluated the role of adjuvant radiation among 53 immunocompromised patients with scalp SCC. Thirty-one were solid organ transplant recipients, 19 had CLL, and 3 had HIV. On pathology, 69.8% had PNI, 24.4% had LVI, and 39.6% had a poorly differentiated tumor. All patients were referred to adjuvant radiation therapy. However, only 45 complied to the recommendation and 8 did not receive the treatment. The three-year DFS among patients who received adjuvant radiation was 80% compared to 62.5% among patients who were treated by surgery alone, and the OS was 62% vs. 37.5% (significance not mentioned).

Manyam et al. [52,56], compared the outcome of surgical and adjuvant radiotherapy treatment for cSCC among immunocompetent and immunocompromised patients, and demonstrated inferior loco-regional control and progression-free survival among the immunocompromised patients, with immunosuppression being an independent risk factor for a worse loco-regional control rate in the multivariate analysis. 

It appears that immunosuppression is indeed a risk factor for worse outcome among cSCC patients, and that these patients seem to experience better outcome when treated with adjuvant radiotherapy, thereby justifying the recommendation of the NCCN to consider adjuvant radiotherapy in these cases. 

## 6. Radiotherapy Combined with Chemotherapy in the Definitive/Adjuvant Setting for Locally Advanced cSCC

There is no high-level randomized controlled evidence in support of the addition of chemotherapy to radiotherapy for locally advanced cSCC. However, this is a common practice, due to the inherent chemosensitivity of these tumors and as an extrapolation from mucosal head and neck SCC. 

In the definitive setting, no high-quality randomized prospective data exist regarding this question, to the best of our knowledge. A small retrospective cohort showed no difference in the outcome with the addition of platinum compounds or cetuximab to definitive intent radiotherapy [61]. With regard to cetuximab as a radiosensitizer (as was prospectively tested in head and neck mucosal, but not cSCC [62]), an 8-patient cohort showed good local control in 5 patients treated, with acceptable toxicity [63].

In the adjuvant setting, recent randomized data are available. TROG 05.01 randomized 321 patients receiving 60–66 Gy of adjuvant radiotherapy in addition to weekly carboplatin or radiotherapy only. This trial demonstrated no benefit from the addition of chemotherapy [64]. However, single-agent carboplatin is not an accepted radiosensitizing regimen, and the cohort may be too small to demonstrate a significant difference. Another prospective trial compared weekly cetuximab with radiotherapy to historical data with a favorable local control profile, with a cost of about 15% grade 3 toxicity [65]. Retrospective data did show a recurrence-free (although not overall) survival advantage for the addition of platinum-based chemotherapy to adjuvant radiotherapy with high-risk lesions (multiple involved nodes, ECE, or involved margins, as an extrapolation from mucosal head and neck SCC) [66]. Another retrospective trial of 30 patients even showed a survival disadvantage to the addition of chemotherapy to adjuvant radiotherapy without a DFS difference, or a difference in acute or chronic radiation toxicity [67].

## 7. Elective Neck Irradiation

The role of elective neck treatment in head and neck cSCC has been a matter of debate [68,69,70]. The first question in order is whether elective treatment—either surgical or by irradiation—is at all necessary among cSCC patients. Amit et al. attempted to examine the role of surgical treatment for cSCC, by describing 1111 patients with head and neck cSCC. Of them, 173 (16%) underwent elective neck dissection, and the rest were observed and surgically salvaged if regional disease appeared. They found no survival benefit among patients treated electively compared with those who were observed and salvaged when cervical metastases appeared. While the overall rate of occult regional spread among those treated electively was 21% (36 of 173), only 5% of patients treated by observation experienced regional recurrence (49 of 938). A multivariate analysis did not demonstrate a survival benefit for the performance of elective neck dissection. These results demonstrate that elective neck treatment is not necessarily associated with survival benefit and should be considered on a case-to-case basis. 

Herman et al. [71] evaluated 107 patients with head and neck cSCC and parotid metastases who were all treated by parotidectomy and either elective neck dissection and adjuvant radiotherapy (N = 42), or only elective neck irradiation (N = 65). They found no difference in the local control rate between the two groups, as one regional recurrence was demonstrated in the elective neck dissection group (2%), and one recurrence was also demonstrated in the elective irradiation group (1.5%). The disease-specific survival was also similar between the groups. These results show that elective neck irradiation can achieve a favorable oncologic outcome, compared to elective neck dissection. 

Wray et al. [72] described 71 patients with head and neck cSCC who were all electively treated with neck irradiation. Indications for elective nodal irradiation included recurrent tumors (32%), a poorly differentiated tumor (35%), perineural invasion (clinical—13%, pathological—76%), a tumor > 2 cm (51%), and immunosuppression background (8.5%). The median radiation dose to the nodal area was 50 Gy. They demonstrated a 96% 5-year regional control rate, with no cases of grade 3 or higher toxicity. 

These results also support the role of radiotherapy as an elective treatment modality among head and neck cSCC patients. The indications for elective neck treatment, however, either surgical or by irradiation, still remain unclear and demand further investigation. 

## 8. Radiation and Immunotherapy

Immune checkpoint inhibitors, especially anti-programmed cell death-1 receptor (anti-PD1) antibodies such as pembrolizumab and cemiplimab, have been approved for the treatment of cSCC not amenable for curative intent treatment with tremendous results [73], and have been evaluated for BCC as a second line in patients who develop resistance to Hedgehog pathway inhibitors [74]. Still, the response to immunotherapy is seen in around 50% of patients [75]. Radiotherapy has a direct effect on tumoral cells because it induces cellular DNA damage. However, in recent years more evidence was gathered on the immunogenic effect of radiation. The damage radiation induces in cancer cells can convert them into an in-situ tumor vaccine by inducing the release of neoantigens during cancer cell death in association with proinflammatory signals that trigger the innate immune system to activate tumor-specific T cells. This is mediated by three important signals: calreticulin, high-mobility group box-1 (HMGP-1), and adenosine triphosphate (ATP) [76]. In addition, radiation’s effects on the tumor microenvironment enhance the infiltration of activated T cells and can overcome some of the barriers to tumor recognition by the immune system. The translocation of calreticulin and other signals to the cell surface promotes the uptake of dying cancer cells by dendritic cells and the release of antigens that can be efficiently presented [77], which could allow for responses in distant nonirradiated sites, namely the abscopal effect [78]. This effect is currently being investigated, with numerous techniques explored in order to augment it, namely hypofractionation, pulsed hypofractionation, and partial tumor volume irradiation.

In a cohort of 33 patients, comprised of 21 patients who received cemiplimab alone and 12 patients who received concomitant radiotherapy and cemiplimab, the objective response rate was 47.6% and 41.6% in the cemiplimab alone and the concomitant radiotherapy and cemiplimab groups, respectively (*p* = 1.000). The disease control rate was 81.8% and 55.6% in the cemiplimab alone and the concomitant radiotherapy and cemiplimab groups, respectively. Concomitant radiotherapy had a real initial beneficial effect because it shortened time to response and improved the 6-month OS. The responses did not seem to be persistent over time in terms of the OS (median OS at 9 months in both groups). It did improve local symptomatology, without increasing the adverse events occurrence [79].

## 9. NMSC Radiation Toxicity

### 9.1. Early-Stage Lesions

1. Acute toxicity—due to the superficial nature of radiotherapy on the one hand, and the targeting of the skin on the other, the main acute toxicity is radiation dermatitis, which may amount to grade 3 toxicity (i.e., wet desquamation). It is typically treated with topical agents and is usually short-lived due to the quick proliferation of cutaneous keratinocytes [80].

2. Late toxicity—the main concern is usually skin necrosis, especially in poorly vascularized areas (scalp, shin), or around skin flaps that have not completely healed at the initiation of radiotherapy. Other concerns are hair loss at the irradiated area, discoloration, and chondronecrosis (mainly in ear or nose lesions). Many papers describing radiotherapy for early-stage lesions do not expressly report late toxicity rates. Those that do range between 0 and 5.7% (for radiotherapy to the pinna) [80].

A caveat of treating early-stage NMSC with radiotherapy is the tendency for multiple lesions in sun-exposed areas (“farmer’s skin”). This may entail metachronous field overlap and increased long-term skin toxicity [81].

### 9.2. Advanced-Stage Lesions

As mentioned above, in advanced-stage lesions—whether treated with definitive or adjuvant intent—a larger and deeper volume is typically irradiated many times with elective treatment to the neck nodal basin and parotid, and therefore the toxicity profile is different. However, mainly due to the unilaterality of radiotherapy and sparing of mucosa, the rate of grade 3–5 adverse events is very low, as little as 0% in some series [72]. However, concurrent systemic therapy (platinum-based or off-label cetuximab) may increase acute dermatitis and mucositis to up to 40% [66,82].

1. Acute toxicity—If the skin is targeted, dermatitis may occur as described above. Elective radiotherapy to the neck may result in mucositis and dysphagia. Radiotherapy to the parotid and course of the facial nerve may result in xerostomia and metallic taste.
2. Late toxicity—While dermatitis and mucositis usually resolve, chronic xerostomia may occur. Tinnitus may occur if the base of the skull is irradiated. Thyroiditis may occur for lower neck radiotherapy, and the acceleration of carotid artery stenosis may also occur.

## 10. Future Directions

Clinicaltrials.gov was searched for active trials involving cSCC and radiotherapy. This search yielded 53 results, with 20 of these trials actively recruiting as of April 2023 and 14 trials having been completed. We present selected trials with a special emphasis on the trials that combine immunotherapy with radiotherapy. 

As immune checkpoint inhibitors are becoming more prevalent as a treatment modality for locally advanced cSCC, studies regarding concurrent treatment with immunotherapy and radiotherapy are attempting to define the efficiency of this combination. A multi-center study by Barker et al. is recruiting patients with T3/T4 unresectable cSCC (defined as such when the surgery is expected to result in significant disfigurement or dysfunction, complete surgical resection is not possible, cases of recurrence after two surgical resections with further curative resection unlikely, or medical background not enabling surgery). The treatment protocol includes two doses of neo-adjuvant cemiplimab in a 3-week interval, followed by concurrent cemiplimab and 70 Gy radiotherapy in 35 fractions over 7 weeks [NCT05574101]. A similar study is attempting to investigate the role of concurrent avelumab (PD-L1 inhibitor) combined with radiotherapy (63–66 Gy over 30 fractions) in the treatment of unresectable cSCC [NCT03737721].

Another prospective study by Lin et al. [83] is evaluating the efficacy and toxicity profile of definitive chemoradiation with immunotherapy (Durvalumab—anti-PD-1 checkpoint inhibitor) for the treatment of advanced-stage (3–4) unresectable cSCC.

These studies can hopefully help consolidate the role and importance of radiotherapy combined with immunotherapy as a leading treatment modality for unresectable cSCC. 

The KEYNOTE-630 study [NCT03833167] is an ongoing randomized control study that compares pembrolizumab with a placebo as adjuvant therapy for patients with locally advanced high-risk cSCC treated with surgery and radiotherapy. The main outcome is recurrence-free survival, with secondary outcomes being OS, quality of life, adverse events, and discontinuation of the treatment due to adverse events. 

Another ongoing randomized controlled study is attempting to define the role of cemiplimab as an adjuvant treatment to surgery and radiotherapy in cases of high-risk cSCC. The study is currently recruiting patients and will compare the effect of adjuvant cemiplimab with a placebo in this setting. The disease-free and overall survival will be compared between the two arms of the study [NCT03969004].

An interesting study by Bossi et al. is investigating the immuno-modulating effect of post-operative adjuvant radiotherapy among patients treated for high-risk cSCC. Their main outcome measure includes a description of DFS for patients whose lymphocyte count 28 days after completion of adjuvant radiotherapy drops to below 500. This study can possibly shed light on the immune-modulating effect of radiotherapy in cSCC patients, and lead to a better understanding of the role of immunotherapy combined with radiotherapy among these patients, as PD-1 immune check-point inhibitors activate lymphocytes in order to allow the patient’s immune system to destroy the cancer cells. 

## 11. Conclusions

Radiotherapy treatment plays an important role in the treatment strategy of NMSC. It can be used both in the adjuvant setting of surgically treated patients with aggressive pathological features, and in the definitive setting, usually when surgery is not performed. Even in an era when immune checkpoint inhibitors are becoming a significant part of the treatment regimen for advanced cSCC, the role of radiotherapy treatment in these cases cannot be undermined, and current and future research may aid in consolidating the role of a combination of these modalities, either concurrently or as adjuvant treatment. 

## Figures and Tables

**Table 1:** Summary of articles describing the role of radiotherapy in the definitive setting in early cutaneous basal and squamous cell carcinoma.

Author	Number of Patients	Pathology	Outcome	Complications	Radiotherapy Modality and Fractionation
Avril 1997 [23]	177	BCC	4 years local control—92.5%	65% dyspigmentation and telangiectasia	Interstitial interventional radiotherapy (brachytherapy)—55% Soft X-ray (contact) radiotherapy—33% Conventional radiotherapy—12%
Locke 2001 [22]	425	T1/T2 BCC + SCC	5 years local control for BCC—94% 4 years local control for SCC—85%	5.8%	Soft X-ray (contact) radiotherapy—60% Electron beam radiotherapy—19% Soft X-ray and electron beam combination—20% Megavoltage—<2%
Schulte 2005 [26]	1267	BCC + SCC	5 years local control for BCC—95.8% 5 years local control for SCC—94%	Hypopigmentation—72.7% Telangiectases—51.5% Ulceration—6.3%	Soft X-ray (contact) radiotherapy—100% 10–100 kV
Barysch 2012 [25]	180	SCC	5 years local control—86.2%		Soft X-ray (contact) radiotherapy—100% 30–60 kV
Cognetta 2012 [24]	1715	BCC + SCC	5 years local control—95%		Soft X-ray (contact) radiotherapy—100% 5 sessions of 7 Gy or 7 sessions of 5 Gy
Bortoluzzi 2022 [27]	56	SCC	5 years local control—71.3%	3%—non-acceptable cosmetic result	Soft X-ray (contact) radiotherapy: 55–60 kV—55% 50–100 kV—39% Other soft X-ray protocol—6%

## Data Availability

Not applicable.
